# Effect of “universal two-child” policy on population changes in Shandong province, China: an interrupted time series analysis

**DOI:** 10.3389/fpubh.2025.1612141

**Published:** 2025-08-28

**Authors:** Keqing Shi, Wenhui Cui, Shuyu Chen, Xueli Zhang, Xin Wang, Mengjun Cao, Hang Gao, Qiang Wang

**Affiliations:** ^1^College of Public Health, Shandong Second Medical University, Weifang, China; ^2^Department of Histology and Embryology, College of Basic Medical Sciences, Shandong Second Medical University, Weifang, China; ^3^College of Clinical Medical Sciences, Shandong Second Medical University, Weifang, China; ^4^Department of Epidemiology, Shandong Second Medical University, Weifang, China

**Keywords:** interrupted time series, the universal two-child policy, fertility rates, births, segmented regression analysis

## Abstract

**Background:**

As population aging intensifies and women’s fertility levels decline continuously, the improvement of fertility policies has emerged as a pivotal concern for most governments. This study aimed to evaluate the effect of the “universal two-child” policy implementation on the birth population trend.

**Methods:**

A quasi-experimental interrupted time series (ITS) study was conducted to analyze the collected data. Data on the birth population of Shandong province from 2000 to 2022 were collected to observe trend changes before and after the intervention.

**Results:**

The birth rate increased immediately in the first year after the intervention (*p* < 0.001), but the trend significantly declined in the following years (*p* < 0.001). After further adjustment for the influence of the pre-fertility policies, urbanization rate, and per capita disposable income, the birth population level still showed a downward trend year by year in the post-intervention period (*p* < 0.001).

**Conclusion:**

The policy implementation helped to boost the population growth in the short term, but the long-term effect of the policy was not optimistic. More targeted incentive strategies should be considered to reverse the continuous decline in fertility rates.

## 1 Introduction

Birth rate indicates the number of live births per 1,000 population occurring at midyear (1). Birth rate is a fundamental demographic indicator that reflects the overall fertility level and population growth potential of a region (2). In 2020, official statistics reported a significant decline in the number of births, with a decrease of approximately 580,000 compared to the previous year. The crude birth rate fell to 10.48 per 1,000 population (3). This downward trend underscores the limited effectiveness of existing fertility policies in reversing the decline, highlighting the persistent challenges faced in stimulating higher fertility rates. The total fertility rate (TFR) was defined as the average number of births during a woman’s life span (4, 5). The fertility pattern was represented by both rates. A study conducted manifested that by 2030, approximately two-thirds of countries (or regions) worldwide will have a TFR below the replacement level of 2.1 (6). Previous studies indicated that the TFR was influenced by education expansion (measured by the proportion of non-student women), marriage delay (measured by the proportion of married women), and the marital fertility rate (7, 8). In 2022, the TFR in China was 1.05, which represents only half of the replacement level (9). These results indicate that the phenomenon of low birth rates may spread rapidly across the world.

The persistently low birth rate poses significant challenges to China’s population structure, economic development, and social stability. Fertility policies, as one of the crucial approaches to improving the demographic structure, could positively encourage population growth. A previous study demonstrated that a long-term decline in fertility rates could cause an imbalance in the demographic structure of a country or a region, gradually transforming from a healthy “pyramid shape” to a “column shape” that symbolized an aging society (10). This structural shift would prompt a country to face a series of social and economic issues, including immense pressures related to older adult care and a shortage of young labor force (10, 11). To address these challenges, China has implemented a series of fertility policies since the 1970s. The one-child policy that was introduced in the late 1970s effectively curbed population growth but also accelerated population aging and exacerbated gender imbalance (12). In response, the government relaxed the selective two-child policy in 2013 and subsequently launched the universal two-child policy in 2016 (13). However, fertility rates have continued to decline despite these policy adjustments, suggesting that non-policy factors such as economic pressures and changing fertility intentions have played a critical role (3). While TFR offers a long-term perspective on reproductive behavior, crude birth rate birth rate serves as a more immediate and data-accessible indicator of population-level fertility trends. Given the availability of official statistics and the aim of evaluating policy effects over time, this study used birth rate as the primary outcome variable for empirical analysis, while referring to TFR for broader contextual interpretation.

Nevertheless, the effectiveness of these interventions in curbing the long-term downward trend in birth rate remains uncertain, as changes in birth rates are shaped by evolving social, economic, and cultural contexts over time (14, 15). A study based on the theory of planned behavior demonstrated that fertility intention was determined by three main factors: fertility attitudes, social fertility norms, and perceived behavioral control over fertility. Meanwhile, socioeconomic and cultural background factors, as well as individual factors, indirectly influenced fertility intentions by affecting these three main factors (10). Additionally, the decline was also linked to the annual decrease in the number of women of childbearing age and the continuing postponement of the age at which women get married and have their first child (16). Some scholars argued in previous studies that the enhancement of women’s sense of independence and the emphasis on cultivating self-worth also contributed to the reduction in fertility (17, 18). A study conducted in Malawian manifested that an increase in women’s income and social status was associated with a reduction in their fertility desire, while women’s individual awareness, income, and status exerted a considerable influence on their reproductive decisions (19). The concept of deliberate childlessness has been accepted by some countries and is continuously spreading (20, 21). In 2021, there were over 600,000 DINK (Dual Income No Kids) families in China, accounting for about 12.4% of the national total, and this population is still growing (22). When the cost consumption associated with fertility behavior imposed a burdensome economic strain on families, many households asserted that their decision not to have children was not a reflection of a lack of parental desire, but rather a consequence of financial constraints (23). In the mid-20th century, China initiated the implementation of the “one-child” policy designed to address the issue of rapid population growth. Unfortunately, although the implementation of this intervention effectively controlled population swell, it also resulted in a series of adverse effects (24). In order to forestall further deterioration of the crisis, China initiated a relaxation of its population control measures, such as the introduction of the “selective two-child” policy (couples were allowed to have a second child if either parent was an only child) at the end of December 2013 (25). However, the fertility intentions of Chinese couples were not stimulated as expected (26). On this basis, China further proposed the “universal two-child” policy, which was implemented simultaneously across the country in early January 2016. The proportion of second children in our country reached 51% in 2017 (27), which was a marked increase compared to the 45% reported in 2016 (28) and the 40.5% recorded in 2015, respectively (11). But the birth rate per year sharply reduced from 13.57‰ in 2016 to 6.77‰ in 2022 (9). However, the effectiveness of policy implementation varies across different regions. Shandong Province was chosen for this study because it is a representative area to examine the impacts of fertility policies in China. Firstly, Shandong has historically maintained relatively high fertility rates compared to many other provinces, reflecting distinct demographic and socio-economic characteristics (29). Secondly, after the implementation of the “universal two-child” policy, Shandong experienced a rapid but short-term rise in birth rates. This was followed by a sharp decline, providing a clear temporal window to assess the policy’s effects. Thus, Shandong offers an ideal balance of demographic stability, data availability, and policy responsiveness to rigorously evaluate fertility policy outcomes in China. In Shandong, the birth rate increased rapidly from 12.55‰ in 2015 to 17.89‰ in 2016 and 17.54‰ in 2017 during the implementation of the “universal two-child” policy. After that, however, the birth rate began to decline sharply, reaching only 6.71‰ in 2022 (30). Previous studies have pointed out that the two-child policy has had limited success in addressing China’s low fertility rate (31). Meanwhile, the policy has had little impact on women’s employment in China, but it has significantly affected the quality of their employment (32). Further research is thus needed to evaluate the effect of the “universal two-child” policy in reversing the ongoing decline in fertility.

To further strengthen the theoretical basis of our study, we draw upon the Theory of Planned Behavior (TPB), which provides a useful framework for understanding fertility intentions in this context. Moreover, to better interpret the determinants of fertility intentions within China’s evolving policy context, this study adopts the TPB as its conceptual framework. According to TPB, fertility decisions are influenced by three key components: individual attitudes toward childbearing, perceived social norms, and perceived behavioral control. These components are further shaped by broader socioeconomic and cultural factors. For example, economic pressure, career goals, societal expectations, and perceived ability to raise children all play a role in shaping fertility preferences and behaviors. This theoretical framework guided the formulation of research hypotheses and the interpretation of empirical findings in this study.

Interrupted time series (ITS) analysis is a quasi-experimental design approach that takes into consideration both longitudinal data and pre-intervention trends and is well-suited for evaluating the long-term effects of population policies such as fertility interventions (33). Therefore, the aim of this study was to use the ITS analysis to estimate the effect of the implementation of the “universal two-child” policy on alleviating low fertility rates in Shandong Province, China. Specifically, this study compared birth population trends before and after the policy implementation to evaluate its effectiveness in addressing low fertility rates in the region.

## 2 Materials and methods

### 2.1 Data source

This study used data from the official statistical yearbooks published by the Shandong Provincial Bureau of Statistics (30). Birth rate data in Shandong Province from 2000 to 2022 were collected. The period 2000–2015 was defined as the pre-intervention phase, and 2016–2022 as the post-intervention phase. Birth rate was selected as the outcome variable due to its clear definition, accessibility and ability to reflect annual demographic trends. The study area was Shandong Province, located in eastern China. In 2016, its birth rate reached the highest level since 2000. Shandong has a large population and showed a strong response to fertility policy changes, making it representative for this analysis. All data were obtained from public sources. No identifiable personal information was involved. Ethical approval was granted by the Ethics Committee of Shandong Second Medical University (Approval No. 2023YX-139). Informed consent was not required.

### 2.2 Model specification and variable definitions

This study employed an ITS design to evaluate the impact of the universal two-child policy on the trend of birth population. The ITS design has been widely used in public policy evaluation and health intervention studies, offering high internal validity and enabling the identification of policy effects without the need for a control group (34). Based on the ITS framework, a segmented regression model was constructed to examine both trend changes and level shifts in birth rates before and after the policy intervention. The model was specified as follows:


(1) $$\begin{array}{l} \text{Variable}\;(\text{birth rate})=\beta_{0}(\text{constant})+\beta_{1}\text{time}\\ +\beta_{2}\text{intervention}+\beta_{3}\text{post}+\varepsilon \end{array}$$

(2) $$\begin{array}{l} \text{Variable}\;(\text{birth rate})=\beta_{0}(\text{constant})+\beta_{1}\text{time}\\ +\beta_{2}\text{intervention}+\beta_{3}\text{post}+\beta_{4}\text{peak}+\beta_{5}\text{urbanization}\\ +\beta_{6}\mathrm{per}\;\text{capita disposable income}+\varepsilon \end{array}$$

In the model, *β*₀ represented the baseline level of birth rate before the intervention, and *β*₁ indicated the annual trend prior to the policy. *β*₂ captured the immediate change in the birth rate following the implementation of the policy, while *β*₃ reflected the change in trend after the intervention. *ε* denoted the error term. Equation 1 served as the basic specification and assessed the direct impact of the universal two-child policy. Equation 2 extended this by adding control variables, including prior fertility policies, urbanization rate, and per capita disposable income, to enhance model interpretability and robustness. *β*₄ measured the potential lagged effect of earlier fertility policies. *β*₅ reflected the impact of urbanization rate. *β*₆ captured the influence of per capita disposable income on birth rate. The overall policy effect was evaluated by the sum of pre- and post-intervention slopes (*β*₁ + β₃). The statistical significance of each coefficient was determined using *p*-values and 95% confidence intervals, where *β*₂ assessed the immediate effect, *β*₃ evaluated the trend change, and *β*₁ + *β*₃ represented the overall policy impact (35).

The variables were defined as follows: time denoted the time variable, coded sequentially from the first year of observation as 0, 1, 2,.., *n*–1, where n was the total number of time points, to capture the trend in birth rate. Intervention was a binary variable, set to 1 in the year of policy implementation and thereafter, and 0 otherwise. Post represented the time trend after the intervention, calculated as the number of years since the intervention, and was set to 0 before the policy. The prior fertility policy variable (peak) reflected the lagged effects of earlier policy changes. These included the two-child policy for couples who were both only children, promoted in 2002, and the selective two-child policy implemented in 2013, which allowed couples with one only-child partner to have a second child (36–38). Based on the birth rate peaks, we selected 2004 and 2014 to represent the delayed impact of these policies. Urbanization indicated the proportion of urban population to total population in each year, reflecting the potential impact of structural changes on fertility levels. Per capita disposable income was measured in thousands of RMB to avoid scale-related estimation bias in the model.

To ensure the validity and robustness of the model, the study accounted for autocorrelation and heteroskedasticity in the time series. When preliminary autocorrelation analysis indicated significant autocorrelation, the Prais–Winsten method was applied to correct the autocorrelated error structure. To further address potential higher-order autocorrelation and heteroskedasticity, Newey–West standard errors were introduced for robust adjustment. In addition, prior fertility policies, urbanization rate and per capita disposable income were included in the model to control for other confounding factors. To examine the robustness of the results, a placebo test was conducted by artificially assigning pseudo-intervention points during 2001–2015, before the implementation of the universal two-child policy, to test whether the model falsely indicated significant effects at non-intervention periods and to rule out confounding from time trend changes. Moreover, considering the possible delay in policy effects, a lag analysis was also performed by setting the intervention points in 2017 and 2018 to assess the delayed impact on birth rates and to comprehensively evaluate the timeliness and dynamic effects of the policy.

### 2.3 Statistical analysis

All data were organized and preprocessed using Microsoft Excel 2019. Statistical analyses and figure plotting were conducted using Stata 15.0 and R version 4.4.1. Descriptive statistics were performed on birth population data before and after the policy intervention, including the mean, standard deviation, and range of birth population and birth rate. An independent-sample *t*-test was used to compare differences between the two periods. A time series plot of the birth rate was generated to preliminarily observe trend changes. The Augmented Dickey–Fuller (ADF) unit root test was used to examine the stationarity of the time series (39). If non-stationarity was detected in the raw series, differencing was applied to obtain a stationary series to meet the basic assumptions of time series analysis. Autocorrelation was assessed using autocorrelation function (ACF) and partial autocorrelation function (PACF) plots. A single-group interrupted time series segmented regression model was employed, with core variables including time, intervention point, post-intervention trend, prior policies, urbanization rate, and per capita disposable income. All statistical tests were two-sided, and a *p*-value less than 0.05 was considered statistically significant.

## 3 Results

### 3.1 Comparison of birth indicators before and after policy intervention

**Figure 1:**
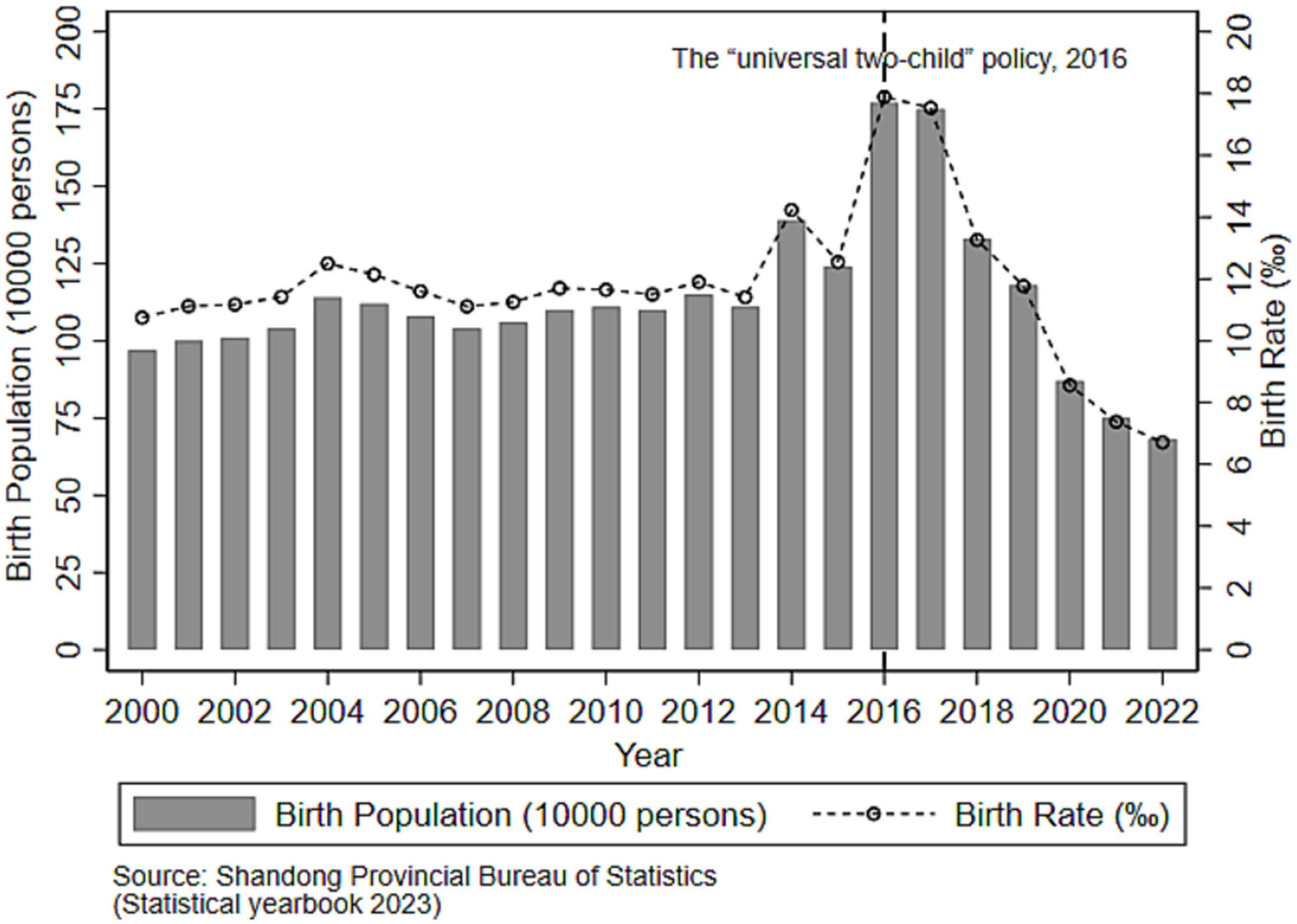
Changes of birth population in Shandong province of China from 2000 to 2022.

As shown in Figure 1 below, the birth population in Shandong province increased rapidly in 2016 and 2017 during the implementation of the “universal two-child” policy, with an increase of 530,000 and 510,000 compared to the number of births in 2015, respectively. Over time, the birth rate showed a downward trend year by year since 2016, specifically reflecting a decline from the highest rate of 17.89‰ to a historic low of 6.71‰ in 2022. At the same time, Figure 1 also shows a significant change in the peaks of the early fertility policy implementation, with the birth rates in 2004 and 2014 being relatively high, exceeding the previous year’s birth rates by 1.08‰ and 2.82‰, respectively. In order to eliminate the deviations from the peak of the early policies implementation in this study (26, 40), the years of 2004 and 2014 were included as control variables in the model, allowing for different potential peaks in birth years.

As shown in Table 1, the average annual birth population in Shandong Province during the post-intervention period (1.1904 million) was higher than that in the pre-intervention period (1.1038 million) and also exceeded the average across the entire study period (1.1301 million). Similarly, the birth rate slightly increased from 11.75‰ before the intervention to 11.87‰ after the intervention, which was marginally higher than the overall average of 11.79‰. Although both indicators showed an upward trend after the intervention, results from the independent samples *t*-test indicated that the differences in birth population (*t* = 0.747, *p* = 0.463) and birth rate (*t* = 0.106, *p* = 0.917) between the pre- and post-intervention periods were not statistically significant.

**Table 1:** Birth population and birth rate per year pre- and post-intervention.

Group	Birth population		Birth rate	
	Mean (SD)	Range	Mean (SD)	Range
Total	113.01 (25.34)	68.22–177	11.79 (2.51)	6.71–17.89
Pre-intervention	110.38 (10.08)	97–139	11.75 (0.83)	10.75–14.23
Post-intervention	119.04 (45.15)	68.22–177	11.87 (4.62)	6.71–17.89
*t*	0.747	–	0.106	–
*p*-value	0.463	–	0.917	–

Pre-intervention dates: 2000–2015; post-intervention dates: 2016–2022.

### 3.2 Stationary analysis of data

**Figure 2:**
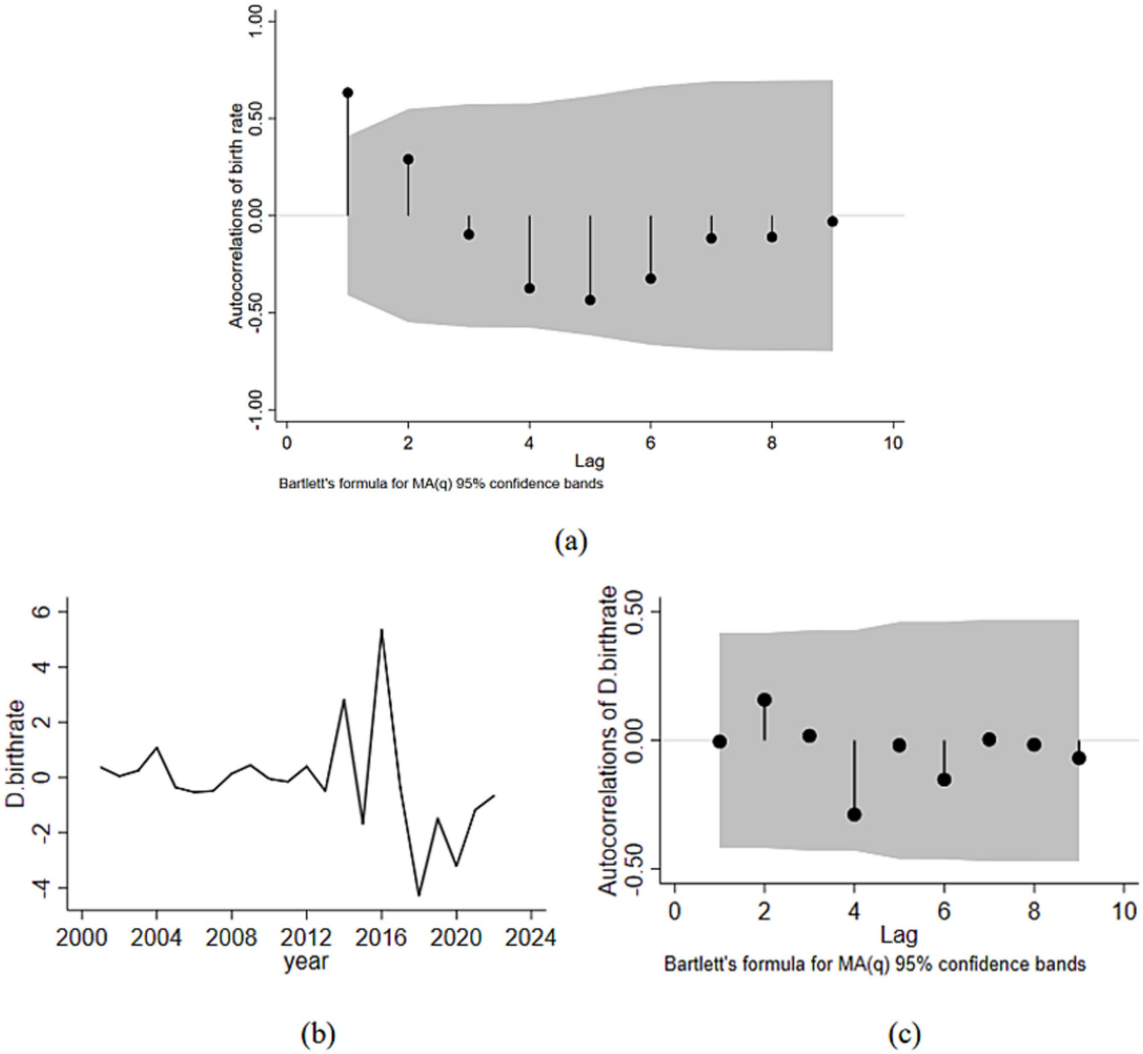
Time series and the ACC of birth rates (the shaded areas are 95% confidence intervals of the ACC). **(a)** Correlogram that showed autocorrelations of birth rate over lags from zero to ten with confidence bands calculated using Bartlett’s formula for MA(q). **(b)** Line graph that indicated changes in D.birthrate from 2000 to 2024. **(c)** Correlogram that showed autocorrelations of D.birthrate over lags from zero to ten with similar confidence bands.

Figure 2 displayed the time series trend and autocorrelations of birth rates. The autocorrelation coefficient (ACC) of the birth rate showed a strong positive correlation only in the initial lag period. At the third lag, the ACC exhibited a negative association, and it was only in the first lag that the ACC significantly exceeded the 95% confidence interval range (see Figure 2a). After taking the first-order difference, the time series plot of the series showed relatively stable fluctuations around the mean, and the autocorrelation plot indicated that the correlation of the series was quite weak throughout the entire lag period, all within the 95% confidence interval (see Figures 2b,c).

Table 2 depicted the stationarity of the time series data. After using the ADF analysis to conduct a unit root test on the series, the results demonstrated that the series was stationary (*p* = 0.001) when the first-order difference of the series was employed. Thus, the first-order autocorrelation in the time series data was observed in this study.

**Table 2:** The stationary test for variables.

Variable	*Z*(*t*)	Interpolated Dickey–Fuller			*p*-value	Test content	Conclusion
		1% critical value	5% critical value	10% critical value			
Birth rate	−1.087	−4.380	−3.600	−3.240	0.931	Tendency	Non-stationary
D.birth rate	−4.615	−4.380	−3.600	−3.240	0.001	Tendency	Stationary

D.birth rate refers to the first-order difference of the birth rate.

When conducting segmented regression analysis, neglecting the issue of serial autocorrelation would lead to false positive results in coefficient statistical tests (33). In this study, the Cochrane–Orcutt iterative Prais–Winsten regression analysis was employed to address the impact of first-order autocorrelation in ITS data. The results of the autocorrelation test for the linear regression model indicated the presence of potential higher-order autocorrelation (*p* < 0.05), as shown in Table 3. Therefore, the Newey–West standard errors analysis was further used to control for potential higher-order autocorrelation and heteroscedasticity.

**Table 3:** The results of regression model autocorrelation test.

Lag	*χ* ^2^	*p*-value	Lag	*χ* ^2^	*p*-value
1	0.105	0.746	6	4.736	0.030
2	0.141	0.707	7	0.378	0.539
3	0.001	0.982	8	1.313	0.252
4	3.932	0.047	9	0.134	0.714
5	0.432	0.515	10	3.541	0.060

### 3.3 Effect of the intervention on the change in the birth population trend

In Table 4, from the results of the segmented regression analysis, this study found that the implementation of the “universal two-child” policy was significantly associated with the change in the trend of the birth population. After applying the Prais–Winsten regression analysis to solve the first-order autocorrelation, the Durbin–Watson statistic was 1.912 compared to the Durbin–Watson statistic of 2.019 using the ordinary linear model, indicating a limited improvement in the first-order serial correlation. After using the Newey–West standard errors analysis, the starting level of the birth rate was estimated at 11.029‰ in baseline period, and the rate appeared a significant increase of 0.096‰ per year before the policy implementation (*t* = 2.31, *p* = 0.032, 95%CI: 0.009–0.183). In the first year after the implementation of the policy (2016), there was an immediate increase in birth rate of 5.580‰ (*t* = 8.13, *p* < 0.001, 95%CI: 4.144–7.016), which corresponded to the period before the policy implementation trend. However, after the implementation of the policy, a long-term treatment effect of the policy found that there was a significantly decrease in the birth rate of 2.188‰ per year (*t* = −13.02, *p* < 0.001, 95%CI: −2.539 ~ −1.836), and the whole effect of the policy indicated that the birth rate was declining at a trend of 2.091‰ per year (*t* = −12.83, *p* < 0.001, 95%CI: −2.433 ~ −1.750). It was worth noting that although the trend coefficient was −2.091, it mainly reflected the short-term decline in the birth rate after the implementation of the universal two-child policy. In fact, the long-term trend of birth rate was influenced by various nonlinear and policy-related factors. Therefore, this result should have been interpreted as a short-term statistical pattern rather than a literal forecast of future birth rates.

**Table 4:** The results of segmented regression analysis on the impact of the “universal two-child” policy implementation on the birth rate.

Birth rate	Coefficient	Estimate	*t*	*p*-value	95%CI
(I) Cochrane–Orcutt iterative Prais–Winsten regression analysis					
Constant	*β*_0_	11.108	26.600	<0.001	(10.231, 11.986)
Time	*β*_1_	0.088	1.910	0.073	(−0.009, 0.185)
Intervention	*β*_2_	5.668	8.170	<0.001	(4.209, 7.126)
Post	*β*_3_	−2.195	−13.980	<0.001	(−2.525, −1.865)
(II) Newey–West standard errors analysis					
Constant	*β*_0_	11.029	37.810	<0.001	(10.418, 11.639)
Time	*β*_1_	0.096	2.310	0.032	(0.009, 0.183)
Intervention	*β*_2_	5.580	8.130	<0.001	(4.144, 7.016)
Post	*β*_3_	−2.188	−13.020	<0.001	(−2.539, −1.836)
Post-intervention linear trend: 2016					
Treated	*β*_1_ + *β*_3_	−2.091	−12.830	<0.001	(−2.433, −1.750)

CI, confidence interval; *R*^2^ = 0.917, *R*^2^_adj_ = 0.904; Durbin–Watson (original) = 2.019, Durbin–Watson (transformed) = 1.912.

When the pre-fertility policies, urbanization rate, and per capita disposable income were included in the analysis, Table 5 showed that early fertility policies (*β*_4_: 1.817, *p* < 0.001, 95%CI: 1.149–2.484) and per capita disposable income (*β*_6_: −0.003, *p* < 0.001, 95%CI: −0.005 ~ −0.002) had statistically significant effects on the birth rate. The segmented regression analysis indicated that the birth rate showed an upward trend before the policy, but the increase was not statistically significant (*β*_1_: 0.025, *p* = 0.416, 95%CI: −0.039 ~ 0.090). In the year of policy implementation, the birth rate increased significantly (*β*_2_: 6.219, *p* < 0.001, 95%CI: 5.745–6.693). After the policy, the birth rate significantly declined by 2.164‰ per year (*β*_3_: −2.164, *p* < 0.001, 95%CI: −2.302 ~ −2.027). Overall, although a short-term increase occurred after the policy, the birth rate continued to show a downward trend (see Table 5).

**Table 5:** The results of segmented regression analysis on the impact of the “universal two-child” policy implementation on the birth rate (includes control variables).

Birth rate	Coefficient	Estimate	*t*	*p*-value	95%CI
Newey–West standard errors analysis					
Constant	*β*_0_	10.144	24.434	<0.001	(9.264, 11.024)
Time	*β*_1_	0.025	0.835	0.416	(−0.039, 0.090)
Intervention	*β*_2_	6.219	27.806	<0.001	(5.745, 6.693)
Post	*β*_3_	−2.164	−33.353	<0.001	(−2.302, −2.027)
Peak	*β*_4_	1.817	5.770	<0.001	(1.149, 2.484)
Urbanization	*β*_5_	2.807	1.959	0.068	(−0.230, 5.844)
Per capita disposable income	*β*_6_	−0.003	−5.401	<0.001	(−0.005, −0.002)
Treated	*β*_1_ + *β*_3_	−2.139	−27.600	<0.001	(−2.290, −1.988)

### 3.4 Robustness analysis

#### 3.4.1 Pseudo-interrupt analysis

This study used a single-group ITS design to evaluate the impact of the large-scale implementation of the “universal two-child” policy on the birth population, but it was challenging to control for the effects of potential confounding factors on the birth population. In this regard, this study employed other pseudo pre-intervention periods (2001–2015) for the interruption test to examine whether the policy intervention still caused an interruption. Supplementary Table 1 showed that the immediate effects of the pseudo-interruption intervention and the long-term effects after the intervention were not statistically significant (*p* > 0.05).

#### 3.4.2 Hysteresis effect analysis

**Figure 3:**
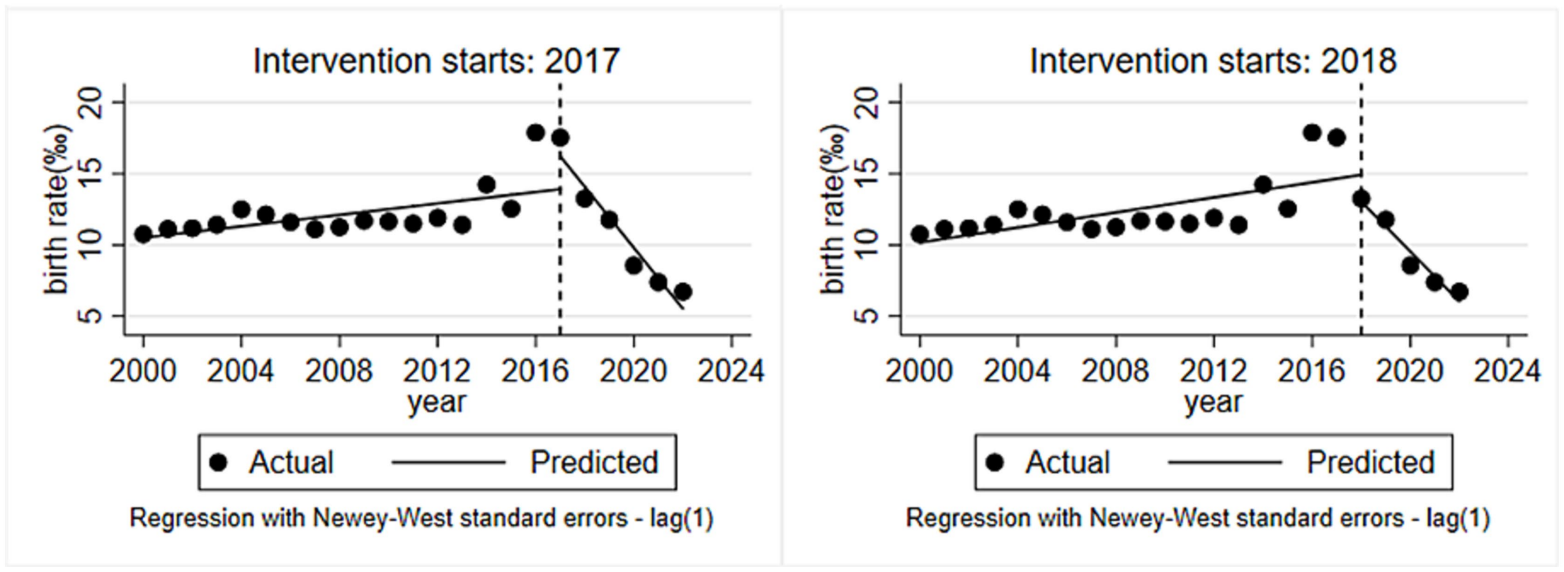
Changes in birth rates before and after lagged interventions.

Considering the lagged effects of the policy implementation, this study selected 2017 and 2018 as the interruption period for the “universal two-child” policy to accurately examine the impact of the policy on the birth population. The results of the hysteresis effect analysis showed that after the implementation of the policy, the birth rate immediately increased by 2.739‰ in 2017 (*t* = 2.908, *p* = 0.010, 95% CI: 0.685–4.782), but the overall birth rate still decreased with a trend of 2.174‰ per year (*t* = −5.740, *p* < 0.001, 95% CI: −2.916 ~ −1.432). Meanwhile, the results with 2018 as the interruption period found that the birth rate immediately declined by 2.692‰, but the difference was not statistically significant (*t* = −1.508, *p* = 0.151, 95%CI: −6.438 ~ −1.054). Subsequently, the birth rate dropped significantly by 2.001‰ per year (*t* = −3.246, *p* < 0.001, 95%CI: −2.395 ~ −0.593) (see Table 6 and Figure 3).

**Table 6:** The results of hysteresis effect analysis of robustness analysis (Newey–West analysis).

Birth rate	Coefficient	Estimate	*t*	*p*-value	95%CI
Interrupt time: 2017					
Constant	*β*_0_	12.399	6.327	<0.001	(8.534, 16.248)
Time	*β*_1_	0.344	1.515	0.149	(−0.131, 0.819)
Intervention	*β*_2_	2.739	2.908	0.010	(0.685, 4.782)
Post	*β*_3_	−2.518	−8.301	<0.001	(−3.167, −1.869)
Peak	*β*4	0.953	1.384	0.185	(−0.304, 1.464)
Urbanization	*β*5	−6.957	−0.875	0.395	(−22.540, 8.626)
Per capita disposable income	*β*6	−0.004	−2.089	0.053	(−0.008, 0.001)
Post-intervention linear trend: 2017					
Treated	*β*1 + *β*3	−2.174	−5.740	<0.001	(−2.916, −1.432)
Interrupt time: 2018					
Constant	*β*_0_	13.614	4.922	<0.001	(7.958, 19.270)
Time	*β*_1_	0.507	1.561	0.138	(−0.173, 1.192)
Intervention	*β*_2_	−2.692	−1.508	0.151	(−6.438, 1.054)
Post	*β*_3_	−2.001	−6.685	<0.001	(−2.588, −1.414)
Peak	*β*4	0.507	0.633	0.536	(−1.180, 2.195)
Urbanization	*β*5	−12.327	−1.081	0.296	(−36.500, 11.787)
Per capita disposable income	*β*6	0.003	1.517	0.148	(−0.001, 0.007)
Post-intervention linear trend: 2018					
Treated	*β*_1_ + *β*_3_	−1.494	−3.246	<0.001	(−2.395, −0.593)

## 4 Discussion

The findings of this study revealed that there was an immediate and significant increase in the birth rate in Shandong Province, China, following the implementation of the “universal two-child” policy. However, the long-term trend indicated that the effectiveness of the policy in promoting population growth was not optimistic. In addition, there was a significant improvement in birth rates during the “universal two-child” policy development period after adjustment for influence of the pre-fertility policies, urbanization rate, or per capita disposable income. The robustness analysis suggested that the effects of the policy on population growth based on the ITS analysis were reliable and further found that the effect of the policy on population growth was short term. Although the differences in birth population and birth rate before and after 2016 were not statistically significant in Shandong Province, this did not contradict our main findings. The birth population increased briefly after the policy was introduced. This suggested a short-term policy effect. Both indicators were influenced by many demographic and socioeconomic factors. Short-term data may not have reflected long-term effects. In addition, the COVID-19 pandemic since 2020 affected fertility behavior and caused a decline in both birth population and birth rate (41). This may have masked the effect of the policy.

The result of the study manifested that after the “universal two-child” policy implementation (2016–2022), there was a significant peak in population births in 2016 and 2017, with birth rates of 17.89‰ and 17.54‰, respectively, which were higher than the birth rate levels among other provinces of China reported by National Bureau of Statistics (i.e., 12.95‰ of national birth rate level in 2016, 9.32‰ of birth rate level in Beijing province in 2016, 15.00‰ of birth rate level in Fujian province in 2017, 13.26‰ of birth rate level in Henan province in 2016, 15.14‰ of birth rate level in Guangxi province in 2017, and 16.00‰ of birth rate level in Tibet in 2017) (9). There were some possible reasons why Shandong has become a top fertility province since the implementation of the “universal two-child policy.” First, the implementation of the policy has allowed the long-accumulated fertility intentions of the individuals in Shandong province to be released in a concentrated manner (42). Second, the country-level economy in Shandong is well-developed, and urban development primarily focused on small and medium-sized cities (30). More importantly, housing prices were much more stable compared to other economically developed places like Beijing, Shanghai and Guangzhou (9). Third, the relatively low cost of raising children may also be one of the main reasons why people in Shandong province were more daring to have children. Conversely, increasing urbanization rates and the concentration of a large population in large cities, resulting in an excessive fertility burden, were the main reasons for birth rates decline in other Chinese provinces (43). However, the birth rates in Shandong have shown a declining trend year by year since 2018, dropping from 13.26‰ in 2018 to 6.71‰ in 2022, which was similar to the trend changes in the birth rates of the country and most provinces (9). The phenomenon suggested that a relaxation of the population control policy has facilitated the accelerated growth of the population, yet the precise effects of the policy on the birth population trend still needed to be further considered.

According to the results of this study, in the first year after the intervention, the birth rate showed a significant immediate increase of 6.219‰ compared to the pre-intervention period. However, the trend declined by 2.164‰ per year in the following years, which was a statistically significant decrease. The results disclosed that while the policy had a notable immediate impact on population growth, its long-term effectiveness was not promising. Compared to the situation of the birth population after the introduction of the pre-fertility policy, this study found that the intercept level has decreased and the upward trend of the birth population has slowed down in the pre-intervention period. The decreasing trend observed in this study suggested that the adoption of pre-fertility policies has played an effective role in increasing the birth population to some extent. However, from the perspective of the long-term effects after the policy implementation, the birth population level still showed a downward trend year after year regardless of whether the pre-fertility policies, urbanization rate, or per capita disposable income were controlled. The rapid downward trend may be associated with the application of family planning policies at different stages, which have caused changes in people’s fertility intentions (26, 42). Furthermore, the introduction of the “three-child policy” in 2021 has not promoted a discernible increase in the birth population, as evidenced by the currently available data (9, 26). The findings in this analysis may be related to the fact that most couples wish or intend to have one or at most two children under the current social environment (42, 44).

The study found that the birth rate rose briefly but then declined under the two-child policy. The brief increase was primarily driven by the release of pent-up fertility intentions among individuals born in the 1970s and 1980s (45). They constituted the main cohort of second-child births during that period. This trend is driven by several deeper underlying factors. A study on fertility costs and benefits pointed out that relative fertility costs included financial pressure, time constraints, opportunity costs and health risks. These factors can influence fertility intentions in various ways. A study on fertility costs and benefits pointed out that relative fertility costs include financial pressure, time constraints, opportunity costs and health risks (46). Firstly, economic pressure has been a important factor that has seriously inhibited the fertility desire of the public because the parenting costs, which consists of the cost of education and housing purchases, are soaring (47). Secondly, as women attain higher levels of education, their opportunity costs tend to rise. More education increases women’s economic opportunity cost of leaving the labor market to care for children and their desire for personal fulfillment (48, 49). Therefore, higher educational attainment of the wife leads to decrease family size. Thirdly, women over the age of 35, especially those over 40, tend to have lower fertility intentions due to the increased risk of adverse pregnancy outcomes (50). Compared with younger women, older mothers face higher risks of chromosomal abnormalities, miscarriage, and preterm birth before 34 weeks of gestation. These factors can influence fertility intentions in various ways. Although implemented under the backdrop of the two-child policy, the birth rate has shown a declining trend in the long term. Another study demonstrated that factors such as age and employment status significantly influence the intention to have a second child among reproductive-aged women in the floating population (51). Although the two-child policy has been widely implemented, the long-term trend shows a general decline in second-child fertility intentions among these women. In fact, many women who already have children find it challenging to balance raising their children and pursuing a career. This is because both responsibilities require a significant amount of time (52). Conflict between work and non-work roles is one of the main sources of stress (53). Therefore, after the implementation of the universal two-child policy, some women choose not to have a second child in pursuit of their career goals. As a result, this may weaken the long-term effectiveness of the two-child policy.

Therefore, the aforementioned researches indicated that while the adjustments to existing fertility policies could simulate population growth in the short term, these were not favorable factors for promoting long-term population growth, which was consistent with the findings of previous research in this field (44, 54). To effectively increase the fertility rate in a country or region, targeted incentive strategies must address specific local economic and cultural factors. In Shandong Province, key economic challenges include the high cost of housing and education and insufficient affordable childcare services. Culturally, traditional expectations of family roles coexist with modern career aspirations, creating pressure especially on women. Therefore, policy recommendations include increasing housing subsidies for young families and expanding accessible, high-quality childcare facilities. Additionally, extending parental leave policies and promoting gender equality and career support for women are crucial. These measures aim to reduce economic burdens and cultural conflicts, thereby encouraging higher fertility rates (43, 55, 56).

There were some strengths in this study. Firstly, this ITS analysis was used in this study to evaluate the effect of the large-scale implementation of the “universal two-child” policy on the growth of the birth population in Shandong province and the result could be taken as a true picture of the fertility situation in the province. Secondly, the difference between the immediate and sustained effects of the two groups after controlling for early population fertility policies, urbanization rate, and per capita disposable income has been evaluated, which could serve as a guideline for relevant governments to design targeted strategies to improve countries’ fertility rates. Thirdly, to overcome the effects of potential confounding factors on the effectiveness of the policy, the pseudo-interrupt analysis and hysteresis effect analysis were further employed to better ensure the robustness and reliability of the sing-group ITS analysis results.

This study had several limitations. Firstly, although the ITS design allowed for quantitative evaluation of policy effects, it was still a quasi-experimental method and could not establish definitive causality. The analysis focused on Shandong Province due to consistent data availability, which provided reliable insight into local fertility trends but limited the generalizability of the findings. Secondly, incomplete data on women’s age groups and urban–rural divisions prevented subgroup analyses, which may have obscured differences in policy effects across populations. Thirdly, when 2018 was used as the interruption point in a supplementary analysis, the immediate effect was not statistically significant, though the long-term trend continued to decline significantly. This may be related to policy lag, annual data granularity, or external environmental factors. Finally, future research will aim to collect higher-resolution data, extend the observation period, and better control for other interventions. It will also incorporate multi-regional panel data and conduct stratified analyses to assess spatial variation and demographic heterogeneity in fertility responses.

## 5 Conclusion

In conclusion, the implementation of the “universal two-child” policy has had a positive short-term effect in alleviating structural population issues and promoting population growth in Shandong Province. However, its long-term effectiveness remains uncertain, as birth rates have continued to decline despite successive policy adjustments. This suggests that the policy alone is insufficient to reverse the downward trend. Firstly, economic pressures—such as the rising cost of living and childrearing—discourage couples from having more children. Secondly, cultural shifts and changing fertility intentions, especially among younger generations, further limit the policy’s impact. To effectively address the low fertility rate, it is essential to explore the underlying causes from economic, social, and cultural perspectives. Future policies should prioritize targeted interventions, including housing support, affordable childcare services, extended parental leave, and stronger gender equality measures, to create a more supportive environment for childbearing.

## Data Availability

The raw data supporting the conclusions of this article will be made available by the authors, without undue reservation.
