# Supplementary material for: Effect of “universal two-child” policy on population changes in Shandong province, China: an interrupted time series analysis
Source: Front Public Health. 2025 Aug 28;13:1612141. doi: 10.3389/fpubh.2025.1612141 (PMC12423096; doi:10.3389/fpubh.2025.1612141)
Supplement: Supplementary file 1 [file Table_1.docx]

| Supplementary Table 1 Robustness test results using pseudo-interruption points | | | | | | |
| --- | --- | --- | --- | --- | --- | --- |
| year | intervention | | | post | | |
|  | estimate | *P*-value | 95%CI | estimate | *P*-value | 95%CI |
| 2001 | 0.670 | 0.482 | (-1.304,2.645) | 0.079 | 0.936 | (-1.981,2.140) |
| 2002 | 1.354 | 0.305 | (-1.355,4.064) | 0.424 | 0.579 | (-1.162,2.010) |
| 2003 | 2.502 | 0.153 | (-1.029,6.033) | 0.817 | 0.295 | (-0.782,2.417) |
| 2004 | 0.739 | 0.822 | (-6.098,7.575) | 0.588 | 0.547 | (-1.437,2.612) |
| 2005 | -1.674 | 0.302 | (-5.003,1.656) | 0.739 | 0.325 | (-0.805,2.283) |
| 2006 | -1.759 | 0.168 | (-4.338,0.821) | 0.657 | 0.379 | (-0.882,2.197) |
| 2007 | -1.756 | 0.073 | (-3.699,0.187) | 0.327 | 0.593 | (-0.945,1.599) |
| 2008 | -1.404 | 0.093 | (-3.068,0.261) | 0.042 | 0.930 | (-0.956,1.040) |
| 2009 | -0.848 | 0.301 | (-2.529,0.834) | -0.139 | 0.731 | (-0.980,0.702) |
| 2010 | -1.174 | 0.214 | (-3.099,0.751) | -0.312 | 0.393 | (-1.065,0.441) |
| 2011 | -1.650 | 0.147 | (-3.946,0.646) | -0.480 | 0.183 | (-1.211,0.250) |
| 2012 | -1.500 | 0.292 | (-4.415,1.415) | -0.618 | 0.125 | (-1.427,0.191) |
| 2013 | -2.661 | 0.125 | (-6.147,0.826) | -0.893 | 0.054 | (-1.802,0.016) |
| 2014 | -1.001 | 0.502 | (-4.088,2.086) | -0.980 | 0.056 | (-1.980,0.020) |
| 2015 | -2.255 | 0.305 | (-6.765,2.255) | -1.436 | 0.052 | (-2.878,0.006) |

*Supplementary Table*
